# Social Determinants of Mental, Physical, and Oral Health of Middle-Aged and Older African Americans in South Los Angeles

**DOI:** 10.3390/ijerph192416765

**Published:** 2022-12-14

**Authors:** Edward Adinkrah, Babak Najand, Arash Rahmani, Najmeh Maharlouei, Tavonia Ekwegh, Sharon Cobb, Hossein Zare

**Affiliations:** 1 Department of Family Medicine, Charles R Drew University of Medicine and Science, Los Angeles, CA 90059, USA; 2 Marginalization-Related Diminished Returns (MDRs) Center, Los Angeles, CA 90059, USA; 3 Mervyn M. Dymally School of Nursing, Charles R Drew University of Medicine and Science, Los Angeles, CA 90059, USA; 4 Department of Health Policy and Management, Johns Hopkins Bloomberg School of Public Health, Baltimore, MD 21205, USA; 5 School of Business, University of Maryland Global Campus (UMGC), Adelphi, Garden City, NY 20783, USA

**Keywords:** middle-aged and older adults, socioeconomic position, socioeconomic status, financial strain, income

## Abstract

Background. A growing body of research suggests that financial difficulties could weaken the protective effects of socioeconomic status (SES) indicators, including education and income, on the health status of marginalized communities, such as African Americans. Aim. We investigated the separate and joint effects of education, income, and financial difficulties on mental, physical, and oral self-rated health (SRH) outcomes in African American middle-aged and older adults. Methods. This cross-sectional study enrolled 150 middle-aged and older African Americans residing in South Los Angeles. Data on demographic factors (age and gender), socioeconomic characteristics (education, income, and financial difficulties), and self-rated health (mental, physical, and oral health) were collected. Three linear regression models were used to analyze the data. Results. Higher education and income were associated with a lower level of financial strain in a bivariate analysis. However, according to multivariable models, only financial difficulties were associated with poor mental, physical, and oral health. As similar patterns emerged for all three health outcomes, the risk associated with financial difficulties seems robust. Conclusions. According to our multivariable models, financial strain is a more salient social determinant of health within African American communities than education and income in economically constrained urban environments such as South Los Angeles. While education and income lose some protective effects, financial strain continues to deteriorate the health of African American communities across domains.

## 1. Background

Oakes and Rossi (2003) define socioeconomic status (SES) as “access to desired resources” [1]. While some measures of SES are objective, other SES indicators are subjective. Some of the pathways that connect SES to health are hypothesized pathways including inadequate money to cover basic needs, access health care, or provide preventive health resources such as acceptable housing or education (Committee on Understanding and Eliminating Racial and Ethnic Disparities in Health Care, 2003).

Although actual income and education are more commonly used as the proxy for SES, financial strain provides additional information about access to SES resources. This is because people with a similar level of education and income may have wide variation in their levels of financial strain. Since inadequacy of income is on the pathway that connects SES to health, it may be preferable to directly measure inadequacy of income to meet an individual’s needs, rather than education and income. To determine inadequacy of income, it is uncommon to have access to the objective data of an individual’s cost of living (e.g., household size, transportation needs) [1,2]. Instead, it is easy to ask for a person’s subjective appraisal of income inadequacy, also known as financial strain [1].

Research has shown that both objective and subjective SES indicators—such as education, income, and financial strain—operate as fundamental causes of health and illness [3,4,5,6,7]. As described by Link, Phelan, Mirowsky, Ross, Hayward, House, Lantz, Williams, and others, these SES indicators protect individuals against a wide range of health problems [3,4,5,6,8,9,10,11,12,13,14,15,16,17]. Education, income, and financial strain are among major social determinants of health in youth [18], adults [19], and middle-aged and older adults [20]. Low education, low income, and severe financial strain partially explain poor health in racial minority groups such as African Americans [21].

Financial strain reflects the economic adversities that individuals experience and report. Perceived financial strain is not addressed with objective and conventional SES indicators, such as education and income, as it is commonly measured as concerns related to a lack of sufficient economic means to afford lifestyle necessities [22,23]. Financial strain is frequently measured using items such as strain in paying for food, clothes, and other bills [24]. Previous research and theoretical work have shown that financial strain is the most salient SES determinant of health among African American individuals [2].

However, the effects of socioeconomic factors such as education, income, and financial strain are not similar across all racial groups [13,14,25]. Financial strain can substantially impact multiple health domains of communities; however, these effects may be more significant for African Americans than White people [24]. The reason for this could be the incredible financial strain experienced by racial/ethnic minorities who reside in communal areas with fewer protective buffers and limited resources [24]. One study suggested that perceived financial strain, not education or income, increased the health vulnerability of African Americans to negative risk factors, such as stress related to discrimination [24]. While SES indicators, such as education and income, reduce perceived discrimination and depression overall [24,26,27,28], these effects are weaker or absent for African Americans who report high perceived discrimination as their education and income increase [28,29,30,31]. Thus, it is essential to study the additive effects of education, income, and financial strain on health among these groups [24]. 

The gradient and threshold effects of education on mortality risk are shown to be diminished for African Americans compared to White people [13,14]. In the presence of racism and discrimination that impacts the education system and labor market, education may provide more oppor 4tunities, such as low-stress, high-paying jobs for White people than for African Americans. Thus, African American individuals with a high level of education may remain at an increased risk of working in worse jobs with high stress and low pay [32,33]. Therefore, under racism and discrimination, highly educated African Americans have worse health than their White counterparts [32,34,35,36]. As these SES indicators differently shape access to resources and exposure to risk factors among racial groups, [28,29,30,31] future research should test the additive effects of education, income, and financial strain on the African American community.

Research has shown weakened economic and health effects of SES indicators, such as education and income, for racialized and marginalized groups, particularly African Americans—a pattern called Minorities’ or Marginalization-related Diminished Returns (MDRs) [32,33,34,35,36,37]. For example, highly educated and high-income African Americans remain at a high risk of poor health [24,32,36,37,38,39,40]. Although this theory is commonly applied for comparative studies, it is also used by within-race studies that find weaker-than-expected effects of SES without having a White control group [41,42]. As White people are an arbitrary control group and should not be regarded as the norm (considering whiteness as the norm is in itself a reflection of racism), within-racial research has its own major implications [43].

These MDRs are robust as they are observed for African American youth [44,45,46,47,48,49,50,51,52,53,54,55,56,57] and adults [24,32,36,37,38,39,40], as well as middle-aged and older adults [58]. As a result of MDRs, middle- and high- SES African Americans show worse-than-expected health. This is because for African Americans, SES indicators such as education and income may have less-than-expected effects, and their financial strain may remain high, so they report poor well-being and health despite middle--to-high SES levels [24,59]. However, very few studies have simultaneously investigated the additive effects of education, income, and financial strain on various aspects of the health of African American middle-aged and older adults in areas that have been historically underserved and under-resourced. 

### Aims

**Figure 1:**
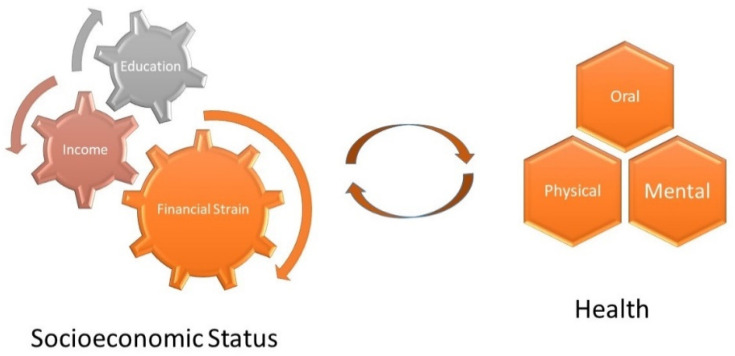
Study conceptual model.

In this study, we investigated the associations between three SES indicators (namely education, income, and financial strain) and three health outcomes (namely poor self-rated mental, physical, and oral health) among African American middle-aged and older adults residing in underserved areas of South Los Angeles. Figure 1 presents the study conceptual model. Underserved areas of South Los Angeles include SPA6, with a population of one million individuals, which has the lowest SES, poor access to health resources, and poor health outcomes.

## 2. Methods

### 2.1. Design 

We conducted this cross-sectional survey in South Los Angeles during the COVID-19 pandemic after vaccination became available for the public during 2021–2022. The study recruited African American middle-aged and older adults primarily from churches and other faith-based centers in South Los Angeles. Data were collected through structured face-to-face interviews on demographic factors; SES characteristics (education and financial strain); living arrangement; marital status; and self-rated mental, physical, and oral health. 

### 2.2. Context of the Study

This survey was conducted in faith-based organizations (i.e., churches) in South Los Angeles during the COVID-19 pandemic after COVID-19 vaccines had become available for the public between 2021 and 2022. This is important in multiple regards. First, African American churches and other faith-based centers play a major spiritual and physical role in African American communities. Second, South Los Angeles and SPA6 is a historically underserved, highly segregated, impoverished, and economically disadvantaged area with high rates of poverty and unemployment among the several racial and ethnic minorities.

### 2.3. Participants and Sampling

The study recruited a convenience sample of middle-aged and older African American adults from faith-based organizations. All participants were Christians, and the faith-based organizations were predominantly Black churches. Eligibility included African American ethnicity, being 55 years or older, and residing in South Los Angeles. Considerable cognitive deficits or participation in a clinical trial were the exclusion criteria in this study. A total number of 150 individuals entered our analysis.

### 2.4. Study Measures

The variables in this study were demographic factors (i.e., sex/gender and age), socioeconomic status indicators (i.e., educational attainment, income, and financial strain), and health (i.e., self-reported mental, physical, and oral health).

#### 2.4.1. Demographic Factors

Gender was a dichotomous variable, with males coded as 1 and females coded as 0. Age was treated as an interval variable (a continuous measure). 

#### 2.4.2. Health Insurance

Only two individuals did not have any health insurance. Thus, we could not include health insurance as a variable. The most common type of health coverage in our sample was Medicare, which was entered as a dichotomous variable in this study as a covariate. This variable was defined as Medicare vs. any other type of insurance or no insurance (*n* = 2). For the sensitivity analysis and robustness check, we ran our models without Medicare in the models and with Medicare, VA insurance, and Medicaid. The results regarding the significance of the effects of SES indicators did not change, so we reported a model that only controlled for Medicare as the most common insurance. This decision was made to avoid overfitting and over-adjustment, given the low sample size.

#### 2.4.3. Education

Education attainment was self-reported and was measured as one of our SES indicators. Education was treated as an interval variable with five levels, with a higher score indicating more education: (1) no high school diploma, (2) high school diploma, (3) some college, (4) bachelor’s degree, and (5) graduate studies.

#### 2.4.4. Income

Income was the 2nd measure of SES and was measured as an interval variable between 1 and 5: A higher score indicated more income. The categories of income were as follows: (1) <USD 10,000; (2) USD 10,000 to 30,000; (3) USD 30,000 to 50,000 (or <50,000); (4) USD 50,000 to 70,000; and (5) more than USD 70,000. For the sensitivity analysis, we tested our results with income as USD <35,000, 35,000–74,999, and >75,000.

#### 2.4.5. Financial Strain

Financial strain was measured using four items in line with Pearlin’s list of main chronic financial strain as our 3rd measure of SES [60,61,62]. These items assessed whether money was sufficient to meet essential needs to pay for food, rent, clothes, and utility bills. These items measured the frequency of not having enough money to buy enough food, clothing, and pay bills. The responses were on a 0 to 5 scale for ‘never’ to ‘always’. We calculated a mean score with a range between 0 and 5. A higher score indicated greater financial strain. We did not include the item on strain in paying medical bills because that item did not load well on the same construct and reduced the reliability from more than 0.9 to about 0.6.

#### 2.4.6. Self-Rated Mental, Physical, and Oral Health

The participants’ self-rated health (SRH) was measured using the conventional single-item health measure commonly used in the literature. This variable has five options ranging from 1 to 5 [63,64,65,66,67,68,69,70,71]. The item reads as “In general, would you say your …… health is: “very good”, “good”, “fair”, “bad”, or “very bad”. The responses were on an interval variable ranging from one to five, with a high score indicating poor self-rated health. Single-item self-rated health measures predict long-term mortality risk [63,64,65,66,67,68,69,70,71]. These questions were repeated for physical SRH, mental SRH, and oral SRH. Previous work has shown that SRH can be used as a continuous measure. As a result, many previous studies have used SRH as a continuous outcome [41,72,73].

### 2.5. Statistical Analysis

In this study, we used Statistical Package for the Social Science (SPSS) 23.0 for the data analysis. First, we described the overall sample by reporting the frequency (n) and relative frequency (%) of our categorical variables and reporting mean and standard deviation (SD) for our continuous measures. Then we ran a Spearman correlation to estimate the bivariate correlations between all study variables. Thus, multi-collinearity was ruled out between education, income, and financial strain. We then ran linear regression models with each health outcome as the dependent variable; education, income, and financial strain as the independent variables; and age, gender, marital status, and living arrangements as confounders. We reported b coefficients, standard error (SE), 95% confidence intervals (95% CI), and p values. For the sensitivity analysis, we ran logistic regression with the following coding of SRH: 0 = “very good”, “good”, and 1 = “fair”, “bad”, and “very bad”. As our results did not change between logistic regression and linear regression, and because linear regression was more parsimonious, we only reported the results of the linear regression model.

### 2.6. Institutional Review Board 

The Charles R. Drew University of Medicine and Science institutional review board (IRB) approved the study protocol. All our participants signed the consent form.

## 3. Results

### 3.1. Descriptive Data

As shown in Table 1, most participants were female (70%), with an average age of 68.5 (SD = 8.6). Of the participants, 48% were on Medicare and 31.3% were living alone. All demographic variables and health data are summarized in Table 1.

### 3.2. Correlations

Table 2 shows the results of the Spearman correlation test. Higher education and income were correlated with lower financial strain (r ranging 0.271 to 0.430, *p* < 0.05). Financial strain was positively correlated with self-rated health outcomes that reflected poor health (r ranging between 0.177 and 0.280, *p* < 0.05). Age and gender were not associated with education, income, or financial strain (*p* > 0.05). 

### 3.3. Regression Results

Table 3 shows the results of three linear regression models, one for each outcome. These models suggested that financial strain, but not education or income, was associated with poor self-rated mental, physical, and oral health. Similar patterns emerged regardless of health outcome (*p* < 0.05 for financial strain and *p* > 0.05 for education and income). The associations were stronger for mental SRH (B = 0.271, *p* = 0.005) followed by oral (B = 0.212, 0.035) and physical SRH (B = 0.175, *p* was only marginally significant). Age was only associated with worse oral SRH (B = 0.035, *p* = 0.005). Gender, living arrangement, and insurance type did not correlate with oral, physical, or mental SRH (*p* > 0.05 for all). 

## 4. Discussion

This study showed that while in a bivariate analysis, education, income, and financial strain are all correlated with poor SRH, in a multivariable analysis, financial strain increases the risk of poor health across domains, while education and income fail to show considerable protective effects. This is important and suggests that, at least in this context and this population, the harm associated with financial strain may be larger than the benefit associated with education and income.

We hypothesized that a high level of education and income would have protective effects and at the same time, that financial strain would pose a risk to the mental, physical, and oral health of African American middle-aged and older adults who reside in historically underserved, highly segregated, impoverished, and economically disadvantaged areas within South Los Angeles. We expected the same pattern across health outcomes [74] for the effects of education, income, and financial strain [75,76,77]. Our multivariable regression models found that, in South Los Angeles, financial strain increases the risk of poor health across domains, while education and income fail to show considerable protective effects. We explain this observation through the impacts of racism on reducing the health gains of education and income [32,34,35,38,39,40,65,78,79,80,81].

Financial strain reflects an understudied aspect of SES among African American communities [24], as it is a predictor for poor health [82] among the general population [83,84] and individuals with a chronic disease [85]. Understudied financial strain is an overlooked SES indicator [86,87,88,89]. Although different studies name the same construct as financial strain, financial stress, economic distress, or economic hardship [90,91,92,93], they all refer to a scarcity of liquid expendable income. Individuals with chronic financial strain cannot use the services that are essential for maintaining their health [83,94].

Financial strain is shown to be one of the most detrimental social determinants of health that operates as a risk factor for a wide range of health outcomes [82,95,96,97,98,99,100,101,102,103]. Additionally, financial strain impacts the health and well-being of the general population [104] and individuals with chronic disease [85]. In the US, African American middle-aged and older individuals with the highest levels of financial strain experience the worst levels of health. Financial strain deteriorates the health of African American middle-aged and older adults in deprived areas where social support and other potential buffers are scarce [84]. Low access to buffers is precarious for the health of the aging African American population [84].

Financial strain limits access to health resources and services [83] and operates as a type of stress [92]. Thus, financial strain increases the risk of chronic diseases [105], including—but not limited to—heart disease [106], diabetes [85], cancer [107], and hypertension [105]. It also worsens self-rated health [82] by causing anxiety [93,108], depression [59,87], and suicide [109]. Financial strain also predicts poor diet [110], smoking [111], and alcohol use [112]. All these increase the morbidity and mortality of populations [25,88,106,107,113]. Thus, the harm associated with financial strain has been widespread and sustained [114,115,116]. Financial strain also causes biological and social decline [92].

In one study in South Los Angeles [117], financial strain predicted chronic disease, chronic pain, self-rated health, depression, and the usage of sick days. In another study, financial strain was a risk factor for poor self-rated health, chronic pain, sick days, chronic disease, and depression, while education failed to show any protective effect for the same community [41]. Similar to the current study, that study compared the role of financial strain with education and showed similar results [41]. 

In our South Los Angeles study, education and income did not show any association with mental, physical, or oral self-rated health. While the weak or non-significant effects of education and income may be because these SES indicators are limited by social stratification [80], financial strain negatively impacts various domains of population health. In highly segregated areas, resources are scarce and support is limited, which may be particularly harmful as older adults age [84]. Previous research has established the detrimental effects of financial strain on the health of African Americans [87,118,119] and middle-aged and older adults [91,94,120], such as its impact on future cardiovascular disease [105]. The harm associated with financial strain in low-resource African American communities is exacerbated by chronic poverty due to unemployment, social isolation, and poor urban community infrastructures, including transportation. Thus, the social factors impacted by financial strain operate as risk factors for various chronic diseases.

Our observation that education and income did not correlate with self-rated mental, physical, and oral health, is in line with MDRs reported for self-rated health [32,37], depression [76,79], and chronic diseases [37,38]. These findings are shown not only for middle-aged and older adults [58], but also for children [37,39,40,80] and adults [32,37,78]. Similar findings are reported from national samples [32,38,78] and local studies [37,81]. Thus, regardless of the age, outcome, and design, studies have shown a reduced effect of education and income and an exacerbated effect of financial strain for African American communities.

In historically under-resourced areas, such as South Los Angeles, the potentially protective effects of education and income on the health and well-being of African Americans is weaker because these areas have limited higher income jobs, healthcare systems, transportation, and healthy food locations, as well as recreational parks for exercise. The financial strain becomes a significant threat to the health and well-being of African American [87,118,119] middle-aged and older adults [91,94,120]. 

Given that financial strain operates as a risk factor, as education and income fail to serve as protective factors, we invite researchers, policymakers, and clinicians to specifically think about the differences across various social determinants of health, from which some may have, and some may not have any effect on the health of African American middle-aged and older adults. As financial necessities operate as a major risk, African American middle-aged and older adults in urban settings may benefit from some cushions that buffer the effects of their financial adversities. Policies that increase the availability of financial supply at the time of need may be a promising strategy to tackle health disparities in African American middle-aged and older adults. We argue that fair lending policies at the time of emergency may be a health policy. Different social determinants have different effects and may not be comparable [121].

This study had some limitations. First, the sampling method was not random. All the participants were recruited from faith-based centers. This particular non-randomized sample can represent a limit. The population that frequents religious places is less heterogeneous than the population itself. Second, the sample size was 150. A larger sample size might show the effect of education and income in our study. However, our previous similar analysis in a larger sample showed similar findings [41]. Third, our study was cross-sectional. In addition, all our study variables were measured at the level of individuals; thus, we cannot claim that education and income do not affect the health of African Americans. We can only claim that these associations were not observed in this sample. We cannot generalize our findings to all African American communities or individuals; thus, we need to interpret the results with caution.

## 5. Conclusions

Financial strain may be particularly detrimental to the health of African American middle-aged and older adults, even those with a higher level of education and income. Although in the bivariate analysis, we could observe the protective effects of education and income, these effects disappeared in the multivariable analysis that included financial strain. According to our multivariable models, in economically deprived areas and constrained settings, the disadvantages of financial strain outweigh the health advantages of education and income. This finding is in line with the literature on MDRs [44,46,122,123,124,125,126,127,128,129,130] which frequently shows that the disadvantages are stronger than the advantages [131]. Diminishing returns of education and income may be due to structural racism, social stratification, and segregation. Policies that reduce the financial strain of African American middle-aged and older adults are essential and may have considerable health returns. Future research should also test the efficacy of economic policies, such as those that increase the availability of cash in times of need on the health outcomes of African American individuals. Such research is particularly important for contexts that are historically underserved and under-resourced. 

## Figures and Tables

**Table 1:** Descriptive Statistics.

	*N*	%
Gender		
Female	105	70.0
Male	45	30.0
Living Alone		
No	103	68.7
Yes	47	31.3
Medicare		
No	78	52.0
Yes	72	48.0
	Mean	SD
Age (Years)	68.53	8.655
Education Level (1–5)	2.8533	1.18371
Income Level (1–5)	2.5986	1.27752
Financial strain (1–5)	1.7217	1.02549
Physical Self-Rated Health (1–5)	2.96	1.026
Mental Self-Rated Health (1–5)	2.28	1.090
Oral Self-Rated Health (1–5)	2.69	1.120

SD: Standard deviation.

**Table 2:** Bivariate correlations (Spearman correlation).

	1	2	3	4	5	6	7	8	9	10
1 Gender (Male)	1.000	−0.064	−0.066	−0.105	−0.030	0.007	0.040	−0.035	0.012	0.006
2 Age (Years)		1.000	0.053	0.380 **	−0.122	0.130	−0.143	0.067	−0.027	0.155
3 Living Alone			1.000	0.013	−0.014	−0.198 *	0.041	0.118	0.120	0.155
4 Health Insurance (Medicare)				1.000	−0.061	0.002	−0.141	0.093	−0.067	0.037
5 Education (1–5)					1.000	0.469 **	−0.271 **	−0.255 **	−0.206 *	−0.152
6 Income (1–5)						1.000	−0.430 **	−0.241 **	−0.299 **	−0.227 **
7 Financial Strain							1.000	0.177 *	0.280 **	0.240 **
8 Physical Self-Rated Health (1–5)								1.000	0.495 **	0.613 **
9 Mental Self-Rated Health (1–5)									1.000	0.609 **
10 Oral Self-Rated Health (1–5)										1.000

* *p* < 0.05; ** *p* < 0.01.

**Table 3:** Summary of three linear regression models with poor self-rated physical, mental, and oral health as outcomes.

	b	SE	Beta	95% CI		*p*
Self-Rated Physical Health						
Gender (Female)	−0.041	0.188	−0.018	−0.413	0.331	0.829
Age (Years)	0.013	0.012	0.104	−0.010	0.036	0.265
Living Arrangement (living alone)	0.216	0.192	0.096	−0.164	0.595	0.263
Medicare	0.071	0.190	0.034	−0.305	0.448	0.709
Education Level (Years)	−0.088	0.089	−0.100	−0.265	0.088	0.323
Income Level	−0.073	0.088	−0.089	−0.248	0.102	0.413
Financial Strain	0.175	0.095	0.170	−0.012	0.362	0.066
Self-Rated Mental Health						
Gender (Female)	0.028	0.189	0.012	−0.344	0.401	0.880
Age (Years)	0.006	0.012	0.049	−0.017	0.030	0.584
Living Arrangement (living alone)	0.179	0.192	0.077	−0.201	0.559	0.354
Medicare	−0.167	0.191	−0.077	−0.545	0.210	0.381
Education (Years)	−0.060	0.089	−0.065	−0.236	0.117	0.503
Income	−0.142	0.089	−0.168	−0.318	0.033	0.110
Financial Strain	0.271	0.095	0.253	0.083	0.458	0.005
Self-Rated Oral Health						
Gender (Female)	0.064	0.198	0.026	−0.327	0.455	0.746
Age (Years)	0.035	0.012	0.261	0.011	0.060	0.005
Living Arrangement (living alone)	0.214	0.202	0.088	−0.185	0.613	0.290
Medicare	−0.083	0.200	−0.037	−0.479	0.312	0.677
Education Level (Years)	0.023	0.094	0.024	−0.162	0.208	0.808
Income Level	−0.151	0.093	−0.171	−0.335	0.033	0.108
Financial Strain	0.212	0.099	0.192	0.016	0.409	0.035

b: Unstandardized regression coefficient; SE: standard error; CI: confidence interval.

## Data Availability

Not applicable.
